# Comparison of hair steroid levels among Wistar rats exposed to different environmental enrichment settings

**DOI:** 10.14202/vetworld.2024.2731-2735

**Published:** 2024-12-06

**Authors:** Alberto Elmi, Niccolò I. Vannetti, Viola Galligioni, Nadia Govoni, Camilla Aniballi, José M. Sánchez-Morgado, Maria L. Bacci, Domenico Ventrella

**Affiliations:** 1 Department of Veterinary Medical Sciences, Alma Mater Studiorum University of Bologna, Bologna, Italy; 2 Department of Veterinary Sciences, University of Pisa, Pisa, Italy; 3 Netherlands Institute for Neuroscience, KNAW, Amsterdam, the Netherlands; 4 Bioresearch and Veterinary Services, The University of Edinburgh, Edinburgh, United Kingdom

**Keywords:** 3Rs, biomarker, environmental enrichment, *Rattus norvegicus*, refinement, steroid, welfare

## Abstract

**Background and Aim:**

Environmental enrichment (EE) is a pivotal tool for creating ideal housing conditions that allow animals to fully display their ethograms. At the micro-environmental level, they should elicit cognitive and social responses and increase physical activities. Hair steroids may be among the best biomarkers to evaluate the effects of prolonged exposure to different enrichments because they are non-invasive and provide information regarding a longer period. This study aimed to compare the hair steroid profiles, specifically corticosterone, cortisol, and dehydroepiandrosterone (DHEA), of Wistar rats exposed to two different EE settings.

**Materials and Methods:**

Twenty (n = 20) outbred Wistar rats were enrolled in this study. First hair collection (T0) was performed 3 days before weaning (at 28 days of life), and then Wistar rats were randomly divided into two equal groups with a sex ratio of 1:1: standard EE group, provided with one rat tunnel, and extra EE group, provided with an additional tunnel hanging from the top of the cage. Environmental conditions were 20°C–24°C, 45%–65% relative humidity, and a 12:12 dark/light cycle, with water and pelleted diet *ad libitum*. The rats were housed in ventilated cages with poplar bedding and nesting material. Hair was sampled again after 3 months (T1). Steroids were quantified using radioimmunoassay or enzyme-linked immunosorbent assay after methanol extraction.

**Results:**

Cortisol was not quantifiable, while corticosterone and DHEA were. After 3 months of exposure (T1), no differences were noted between the experimental groups. On data categorization per sex, females showed higher levels of all steroids than males. In males, the extra EE group had higher corticosterone levels.

**Conclusion:**

This study showed that corticosterone and DHEA are quantifiable in rats’ hair, yet bigger datasets are needed to better understand the physiological levels of these hormones in such a matrix. Different enrichment settings induced differences between and within sex.

## Introduction

Rats (*Rattus norvegicus*) are among the most common laboratory animal species, and they can be used for a wide variety of experimental protocols and research purposes [1]. The use of *in vivo* animal models for biomedical research should always imply the highest welfare standards, not only for ethical and legislative reasons but also for scientific reliability. It is now recognized how housing settings strongly impact the welfare of livestock and animals used in biomedical research, thus producing high-quality scientific data [2] and their reproducibility and scientific reliability. In other words, good husbandry practices are essential to ensure the optimal performance and health of experimental animals. These include adequate nutrition, enrichment and training, proper handling, and good housing conditions. Environmental enrichment (EE), both social and physical, represents a pivotal tool for creating housing conditions that allow animals to fully display their ethograms and should elicit cognitive/social responses and increase physical activities [3]. For rats, as in most social laboratory species, both social and physical enrichment is essential. Thus, animals must be provided with adequate EE as part of a comprehensive animal welfare program [4], with an attempt to vary them as much as possible because animals tend to lose interest in the same objects over time.

According to Neville *et al*. [5], rats prefer high-complexity cages with several types of enrichment. Different types of enrichments, as well as their presence or absence, affect the stress response of the animals and the cagemate relationship. Because they can modulate stress levels and social interactions, EEs may affect endocrine profiles, considering the strong relationship between behavior and steroid hormones [6]. Hair appears to be the best matrix to evaluate steroid levels over a longer period because it accumulates hormones within its shaft [7]. Glucocorticoids are the most commonly used steroids for welfare assessment because stressors increase adrenal secretion [8]. Despite cortisol being the key stress hormone in most mammals, corticosterone is the most important stressor in rodents and can be quantified in plasma [9], urine [10], saliva [11], feces [12], and hair [13]. Another steroid indicative of well-being is the androgen precursor dehydroepiandrosterone (DHEA), a sexual hormone with anti-glucocorticoid and neuroprotective capabilities, also known to reduce agonistic interactions in humans [14]. Quantifying glucocorticoids in animals’ hair may represent a pain-free tool for assessing the long-term effects of exposure to different EEs, as a potential retrospective biomarker of long-term hypothalamic-pituitary-adrenal axis activity [15]. In addition, it is not influenced by the characteristic pulsatile release pattern of steroids into the bloodstream.

This study aimed to compare the hair steroid profiles, specifically corticosterone and DHEA, of Wistar rats exposed to two different EE settings. In this study, cortisol was also investigated. This preliminary study could help further our understanding of the importance of using more complex environmental settings in rats through physiological biomarkers. In addition, it should increase current knowledge regarding the role of DHEA as a biomodulator of well-being and welfare.

## Materials and Methods

### Ethical approval

This study relies on opportunistic hair samplings, which are not classified as procedures according to the European Directive 2010/63/UE; therefore, no ethical approval was needed. All animals were kept according to the European Union Regulations (S.I. No. 543 of 2012) [16], following the Commission Recommendation on Guidelines for the Accommodation and Care of Animals Used for Experimental and Other Scientific Purposes [17].

### Study period and location

The study was conducted from February to May 2021 at the Comparative Medicine Unit of the Dublin Trinity College. Hair samples were shipped to the Department of Veterinary Medical Sciences (DIMEVET) of the Alma Mater Studiorum - University of Bologna for hormonal quantifications.

### Animals

Twenty (n=20) Tcdi:WI(Han) Wistar rats born and housed within the Comparative Medicine Unit at Dublin Trinity College were included in the study. Rats were housed in individually ventilated cages (GR 1800 Double Decker for breeding and 2000P for stock animals, Tecniplast, Buguggiate, Italy) on poplar bedding (Select, JRS, J. Rettenmaier & Söhne GmbH + Co KG, Germany), with two pieces of nesting material (Bed- r’Nest, Dates and Ltd, Manchester, UK), and a red rat tunnel (Dates and Ltd, Manchester, UK) (height: 80 mm; length: 150 mm), as standardized EE per cage. Water was supplied through reverse osmosis-filtered automatic watering (Triple Red, Buckinghamshire, UK). Rats were fed a standard irradiated pelleted diet *ad libitum* (LabDiet PicoLab Rodent Diet 20 5053, LabDiet, Missouri, USA) within a range of 25.0–50.0 kGy. Rats were separated by sex at weaning (28 post-natal days) and were housed in groups. The environmental conditions were 20°C–24°C, 45%–65% relative humidity, and a 12:12 dark/light cycle. The colony was screened every 3 months according to the Federation of European Laboratory Animal Science Associations (FELASA) recommendations and was found free of all listed agents [18].

### Experimental design

For the trial, Wistar rats were randomly divided at weaning (28 days) into two groups: Standard EE (n = 10, sex ratio 1:1) provided with one rat tunnel and extra EE (n = 10, sex ratio 1:1) provided with an additional tunnel hanging from the top of the cage. Hair was preliminary collected for analysis 3 days before group allocation (T0, 25 days of life) and then 3 months after grouping (T1). The second sampling time was chosen to capture the effects of new housing conditions and to allow adequate hair regrowth, considering that hair begins to grow from 2 to 6 days after shaving [19]. Hair was collected from the back area using electric clippers and stored at 4°C in the dark. Steroid extraction was performed as described by Elmi *et al*. [13] and Bacci *et al*. [20]. Briefly, the samples were washed in bi-distilled water and isopropanol, minced to ensure a larger surface area, and then added with methanol for overnight extraction. On centrifugation, a fixed amount of the supernatant was recovered in a bacteriological glass and evaporated to dryness under a chemical hood. Dry extracts were then recovered with a fixed volume of aqueous solvent and stored at −20°C until analysis. The levels of DHEA and cortisol were quantified using the radioimmunoassay, as described by Elmi *et al*. [13]. Corticosterone was measured using a commercial enzyme-linked immunosorbent assay (ELISA) kit (Corticosterone Competitive ELISA kit, Thermo Fisher Scientific, Life Technologies Corporation, MD, USA).

### Statistical analysis

Statistical analysis was performed using GraphPad Prism 9.0 (GraphPad Software Inc., San Diego, CA, USA). Shapiro–Wilk test was used to assess the normality of data distribution. Welch’s t-test was performed to compare hormone levels between groups. Data of T1 were analyzed by a two-way analysis of variance (ANOVA) followed by Tukey’s *post hoc* multiple comparison test. Significance was set at p < 0.05.

## Results

Cortisol was not quantifiable regardless of the samples. Corticosterone and DHEA levels per time point and sex are presented in Table-1. Hormonal quantification on T0 samples, usually discharged as they refer to a pre-experimental period, was also performed mainly to rule out potential biases to the study created by the extremely delicate weaning period. Both corticosterone and DHEA levels at T0 were extremely consistent after sex categorization of the data (males vs. females; corticosterone: p = 0.9542; DHEA: p = 0.8623).

**Table-1:** Descriptive analysis of corticosterone and DHEA levels according to time point and sex.

Timepoints and groups	Corticosterone pg/mg	DHEA pg/mg
T0		
♂ (n = 10)	440.53 ± 134.22	52.22 ± 22.06
♀ (n = 10)	444.13 ± 151.64	54.77 ± 21.58
T1		
Standard EE		
♂ (n = 5)	148.67 ± 19.55	14.42 ± 2.82
♀ (n = 5)	365.89 ± 95.95	52.46 ± 15.37
Extra EE		
♂ (n = 5)	269.27 ± 58.75	15.21 ± 3.53
♀ (n = 5)	380.40 ± 53.39	31.33 ± 7.67

DHEA=Dehydroepiandrosterone, Standard EE=Standard environmental enrichment (1 tunnel on the bedding), Extra EE=Extra environmental enrichment (additional tunnel hanging)

For the T1 samples, the results of the two-way ANOVA used to analyze DHEA (Table-2), highlighted a statistically relevant difference (p = 0.004) when comparing males versus females, but not for the two different enrichment settings nor for the interaction between the variables.

**Table-2:** Relevant *post hoc* Tukey’s multiple comparison test results at T1 for DHEA.

Groups	p-value
Standard EE ♀ versus standard EE ♂	0.0161
Standard EE ♀ versus extra EE ♂	0.0123
Extra EE ♀ versus standard EE ♂	0.0473

DHEA=Dehydroepiandrosterone, Standard EE=Standard environmental enrichment (1 tunnel on the bedding), Extra EE=Extra environmental enrichment (additional tunnel hanging)

For corticosterone instead (Table-3), both sex (p = 0.0008) and EE (p = 0.0278) were statistically relevant, but not their interaction.

**Table-3:** Relevant *post hoc* Tukey’s multiple comparison test results at T1 for corticosterone.

Groups	p-value
Standard EE ♀ versus standard EE ♂	0.0240
Extra EE ♀ versus standard EE ♂	0.0027
Extra EE ♀ versus extra EE ♂	0.0494
Standard EE ♂ versus extra EE ♂	0.0179

Standard EE=Standard environmental enrichment (1 tunnel on the bedding), Extra EE=Extra environmental enrichment (additional tunnel hanging)

**Figure-1:**
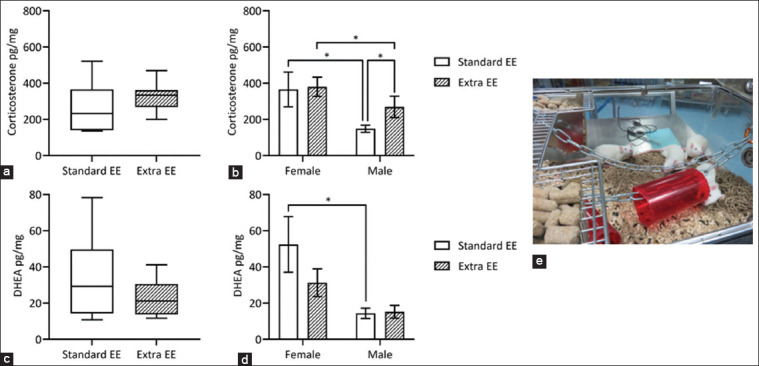
Graphical representation of the results at T1, and an image of the Extra EE setting. (a) Corticosterone Welch t-test, (b) Corticosterone ANOVA, (c) DHEA Welch t-test, (d) DHEA ANOVA, and (e) Extra EE setting; *p < 0.05. EE=Environmental enrichment, ANOVA=Analysis of variance, DHEA=Dehydroepiandrosterone.

The results of 3 months of exposure to different environmental settings (T1) are presented in Figure-1.

Welch’s t-test showed no differences between the groups (Figure-1a, corticosterone p = 0.1850; Figure-1c, DHEA p = 0.2181). Corticosterone ANOVA (Figure-1b) highlighted the effects of sex (p = 0.0008) and enrichment setting (p = 0.0278): Differences were recorded between males and females both within the Standard EE (p = 0.0240) and Extra EE (p = 0.0494) groups, alongside a difference between Standard EE males and Extra EE males (p = 0.0179). In the DHEA ANOVA (Figure-1d), only sex influenced the data (p = 0.0004), with a difference between females and males in the standard EE group (p = 0.0161).

## Discussion

This study aimed to evaluate the effects of exposing laboratory rats to different EE settings by analyzing hair steroids as a non-invasive tool for welfare assessment. The key hypothesis is that a positive effect on the physiologic conditions of animals, resulting from the introduction of an additional enrichment, would result in increased DHEA and potentially lower corticosterone levels. The underlying concept is that laboratory animals like rats prefer more complex and renovated environments that allow them to display their natural behavioral patterns [21]. In particular, the animals enrolled in the present study were supplied with either a single rat tunnel placed on the bedding or an additional one hanging from the top of the cage. The rationale for the addition of a hanging tunnel was driven by the typical climbing behavior of rats without actually compromising the bedding and floor space provided to the animals. Similarly, the age of the animals and the time-placement of the trial were chosen as the most feasible since weaning is the perfect moment to rearrange animals into new groups, also accounting for sex. The preliminary nature of the study and the opportunistic nature of the sampling make for a relatively small sample size, which may be considered the study’s main limitation.

The hormones selected were corticosterone and cortisol, representative of the stress-related hypothalamic-pituitary-adrenal axis, and DHEA, which is involved in sexual behavior on hypothalamic-pituitary–gonadal axis modulation. Literature seems to agree on the predominant role of corticosterone over cortisol in rats, and the results of our study confirmed cortisol to be non-quantifiable. As expected, cortisol was not quantifiable. Indeed, the absence of 17-hydroxylase (CYP17) in the adrenocortical zona fasciculata enzyme prevented rats and mice from producing significant cortisol [22]. No difference was recorded in T0 (3 days before weaning), suggesting that the selected population was homogenous before being randomly allocated to the groups. This time point is important for ensuring that only regrowth occurring during the experimental phase was analyzed. We also decided to shave the animals before weaning to avoid stress due to the separation of the mother. Regardless of the experimental group, corticosterone hair levels at T1 differed between sexes, with higher levels observed in females.

A previous study by Scorrano *et al*. [15] indicates that female rats usually show higher plasma corticosterone levels, but few data are available on hair corticosterone. In male rats, corticosterone levels were significantly higher in animals with the additional tunnel. This finding may seem to contradict the original hypothesis, but it may not. A potential explanation for this result may be hierarchical competition between males and the use of the additional tunnel hanging from the top of the cage. This could support that rats prefer complex ambit enrichments over simple ones; preference studies are needed to determine differences between the two sexes to obtain a more complete situation. Nonetheless, despite such differences, male corticosterone levels were always lower than females. Due to the lack of hair corticosterone reference intervals, it is difficult to understand whether the corticosterone levels observed were physiological. Regarding the quantification of DHEA in rats’ hair, our study is one of the few reports available in literature [23, 24]. No differences were found between the experimental groups, but females had higher EE levels within the standard EE group than males. Despite the lack of statistical significance, extra EE females had lower values than standard EE females. Once again, interpreting these data can be challenging due to the lack of previous studies and the plethora of biological functions exerted by DHEA.

## Conclusion

Overall, this study showed that corticosterone and DHEA are quantifiable in rat hair, as opposed to cortisol. The addition of an extra rat tunnel induced an increase in male corticosterone levels, potentially due to hierarchical competition for usage of the tunnel itself. Females always showed higher hair steroid levels than males. A larger dataset will be needed to better understand the physiological levels of these hormones in hair, especially considering the relatively high individual variability. This study is preliminary and relied on opportunistic sampling, making for a relatively low sample size, which, according to the authors, represents the key limitation of the study. In addition, rats easily become accustomed to the presence of given enrichments, and rotation is often encouraged as a novelty factor. Nonetheless, based on these results, future studies with a dedicated design may help provide more information regarding the behavior and husbandry of rats using hair as a less invasive matrix for quantifying hormones.

## Authors’ Contributions

AE: Conceptualization, formal analysis, and original draft preparation. NIV: Methodology and original draft preparation. VG: Conceptualization and methodology. NG: Methodology and validation. CA: Methodology. JMSM and MLB: Supervision. DV: Conceptualization, formal analysis, and draft review and editing. All authors have read and approved the final manuscript.

## References

[1] Tunstall B.J, Vendruscolo L.F, Allen-Worthington K, Suckow M.A, Hankenson F.C, Wilson R.P, Foley P.L (2020). Rat models of alcohol use disorder. The Laboratory Rat.

[2] Sherwin C.M (2004). The influences of standard laboratory cages on rodents and the validity of research data. Anim. Welfare.

[3] Ismail T.R, Yap C.G, Naidu R, Pamidi N (2021). Enrichment protocol for rat models. Curr. Protoc.

[4] Hubrecht R.C, Carter E (2019). The 3Rs and humane experimental technique:Implementing change. Animals (Basel).

[5] Neville V, Lind J, Mendl E, Cozma N.E, Paul E.S, Mendl M (2023). A mapping review of refinements to laboratory rat housing and husbandry. Lab. Anim. (NY).

[6] Bell M.R (2018). Comparing postnatal development of gonadal hormones and associated social behaviors in rats, mice, and humans. Endocrinology.

[7] Ventrella D, Elmi A, Bertocchi M, Aniballi C, Parmeggiani A, Govoni N, Bacci M.L (2020). Progesterone and cortisol levels in blood and hair of wild pregnant red deer (*Cervus elaphus*) hinds. Animals (Basel).

[8] Oyola M.G, Handa R.J (2017). Hypothalamic-pituitary-adrenal and hypothalamic-pituitary-gonadal axes:Sex differences in regulation of stress responsivity. Stress.

[9] Mucignat-Caretta C, Cavaggioni A, Redaelli M, Da Dalt L, Zagotto G, Gabai G (2014). Age and isolation influence steroids release and chemical signaling in male mice. Steroids.

[10] Thorpe J.B, Rajabi N, Decatanzaro D (2012). Circadian rhythm and response to an acute stressor of urinary corticosterone, testosterone, and creatinine in adult male mice. Horm. Metab. Res.

[11] Nohara M, Tohei A, Sato T, Amao H (2016). Evaluation of response to restraint stress by salivary corticosterone levels in adult male mice. J. Vet. Med. Sci.

[12] Auer K.E, Kußmaul M, Möstl E, Hohlbaum K, Rülicke T, Palme R (2020). Measurement of fecal testosterone metabolites in mice:Replacement of invasive techniques. Animals (Basel).

[13] Elmi A, Galligioni V, Govoni N, Bertocchi M, Aniballi C, Bacci M.L, Sánchez-Morgado J.M, Ventrella D (2020). Quantification of hair corticosterone, DHEA and testosterone as a potential tool for welfare assessment in male laboratory mice. Animals. Basel.

[14] Maninger N, Wolkowitz O.M, Reus V.I, Epel E.S, Mellon S.H (2009). Neurobiological and neuropsychiatric effects of dehydroepiandrosterone (DHEA) and DHEA sulfate (DHEAS). Front. Neuroendocrinol.

[15] Scorrano F, Carrasco J, Pastor-Ciurana J, Belda X, Rami-Bastante A, Bacci M.L, Armario A (2015). Validation of the long-term assessment of hypothalamic-pituitary-adrenal activity in rats using hair corticosterone as a biomarker. FASEB J.

[16] European Union (Protection of Animals Used for Scientific Purposes) Regulations 2012 (S.I. No. 543 of 2012). https://www.irishstatutebook.ie/eli/2012/si/543/.

[17] Commission Recommendation of 18 June 2007 on Guidelines for the Accommodation and Care of Animals Used for Experimental and Other Scientific Purposes (Notified under Document Number C(2007) 2525) (Text with EEA Relevance) (2007). https://eur-lex.europa.eu/legal-content/GA/TXT/?uri=CELEX:32007H0526.

[18] Erratum to “FELASA recommendations for the health monitoring of mouse rat, hamster, guinea pig and rabbit colonies in breeding and experimental units” (2015). Lab. Anim.

[19] Liu L.Y, Guo D.S, Xin X.Y, Fang J (2008). Observation of a system of linear loops formed by re-growing hairs on rat skin. Anat. Rec. Hoboken.

[20] Bacci M.L, Nannoni E, Govoni N, Scorrano F, Zannoni A, Forni M, Martelli G, Sardi L (2014). Hair cortisol determination in sows in two consecutive reproductive cycles. Reprod. Biol.

[21] Chrzanowska A, Modlinska K, Goncikowska K, Pisula W (2022). Rat's response to a novelty and increased complexity of the environment resulting from the introduction of movable vs. stationary objects in the free exploration test. PLoS One.

[22] Raff H (2016). CORT, Cort, B, corticosterone, and now cortistatin:Enough already!. Endocrinology.

[23] Peng F.J, Palazzi P, Viguié C, Appenzeller B.M.R (2022). Hormonal profile changes induced by pesticide mixture exposure in female rats revealed by hair analysis. Chemosphere.

[24] Peng F.J, Palazzi P, Viguié C, Appenzeller B.M.R (2022). Measurement of hair thyroid and steroid hormone concentrations in the rat evidence endocrine disrupting potential of a low dose mixture of polycyclic aromatic hydrocarbons. Environ. Pollut.

